# Establishment of an accurate and fast detection method using molecular beacons in loop-mediated isothermal amplification assay

**DOI:** 10.1038/srep40125

**Published:** 2017-01-06

**Authors:** Wei Liu, Simo Huang, Ningwei Liu, Derong Dong, Zhan Yang, Yue Tang, Wen Ma, Xiaoming He, Da Ao, Yaqing Xu, Dayang Zou, Liuyu Huang

**Affiliations:** 1 Institute of Disease Control and Prevention, Academy of Military Medical Sciences, Beijing 100071, China

## Abstract

This study established a constant-temperature fluorescence quantitative detection method, combining loop-mediated isothermal amplification (LAMP) with molecular beacons. The advantages of LAMP are its convenience and efficiency, as it does not require a thermocycler and results are easily visualized by the naked eye. However, a major disadvantage of current LAMP techniques is the use of indirect evaluation methods (e.g., electrophoresis, SYBR Green I dye, precipitation, hydroxynaphthol blue dye, the turbidimetric method, calcein/Mn^2+^ dye, and the composite probe method), which cannot distinguish between the desired products and products of nonspecific amplification, thereby leading to false positives. Use of molecular beacons avoids this problem because molecular beacons produce fluorescence signals only when binding to target DNA, thus acting as a direct indicator of amplification products. Our analyses determined the optimal conditions for molecular beacons as an evaluation tool in LAMP: beacon length of 25–45 bp, beacon concentration of 0.6–1 pmol/μL, and reaction temperature of 60–65 °C. In conclusion, we validated a novel molecular beacon loop-mediated isothermal amplification method (MB-LAMP), realizing the direct detection of LAMP product.

Established in 2000^1^, loop-mediated isothermal amplification (LAMP) is an *in vitro* DNA amplification technique using 4–6 different primers to identify 6–8 distinct target sequences. This method uses a DNA polymerase with strand displacement activity and yields large quantities of DNA in less than an hour under constant temperature (thus obviating the need for expensive thermocyclers). Moreover, a correspondingly large amount of white magnesium pyrophosphate precipitate is synthesized as a by-product, enabling direct visual confirmation of amplification success through monitoring of mixture turbidity. These characteristics provide results in a fast, efficient, and cost-effective method that theoretically should amplify target sequences with higher specificity than PCR. Since its introduction, LAMP has seen wide interdisciplinary application, for example in detecting microorganisms (e.g., bacteria^2^,^3^, fungi^4^, and viruses^5^,^6^) diagnosing genetic diseases^7^, and even early sex determination^8^.

A major disadvantage of LAMP, however, is the increased likelihood of false positives. Although the recognition of 6–8 independent target sequences (via 4–6 primers) suggests higher specificity and sensitivity than standard PCR, which uses only two primers, the increase in primers can also cause nonspecific amplification^9^. Compounding this problem is the fact that traditional methods of evaluating LAMP reactions cannot directly check for nonspecific amplification, among other issues. Below, we give a brief overview of the most common techniques for LAMP detection and some associated disadvantages.

The first technique, SYBR Green I staining^10^, is based on nucleic acid dyes, which could be excited by ultraviolet light after binding to DNA and added directly to the LAMP product for determining reaction success. However, SYBR Green I binds to any double-stranded DNA, including non-target sequences, resulting in high background values. Both hydroxynaphthol blue dye (HNB)^10^ and calcein/Mn^2+ ^11 are metal ion indicators that can change color when Mg^2+^ is consumed in the reaction solution, they fail to assess whether the LAMP reaction has successfully amplified the target sequences. Turbidity^12^ measurement use magnesium pyrophosphate precipitation for estimation, which is currently the most accepted method for evaluating LAMP reactions.

In summary, the indirect nature of existing detection methods signifies a major shortcoming of LAMP technology. In this study, we therefore developed and validated a method to detect LAMP products directly. Both TaqMan and molecular beacons were under consideration for direct validation of amplification, but we chose to use molecular beacon probes because, in contrast to TaqMan, they are not dependent on Taq exonuclease activity, which many DNA polymerases do not possess. Based on insights from fluorescence quantitative PCR technology, we named our technique the molecular beacon loop-mediated isothermal amplification (MB-LAMP). Molecular beacons (MBs) are fluorescent nucleic acid probes with a hairpin structure, developed in 1996^13^. The hairpin structure prevents fluorescence because the quencher is physically close to the fluorophore. However, binding to the LAMP reaction product sequences alters MB spatial configuration, separating the fluorophore and the quencher at both ends of a single strand of nucleic acids and thereby desorbing fluorescence (Fig. 1). In conditions of LAMP, i.e., a high betaine concentration (0.8 M in a 25 μL reaction mixture) at 60–65 °C, double-stranded DNA maintains a dynamic balance in the state of half dissociation. This gives MBs the opportunity to hybridize with the amplification products directly, opening the hairpin structure and emitting a detectable fluorescent signal. Because a fluorescence signal cannot be generated without the target amplification product, our method solves the problem of nonspecific amplification.

## Results

### Length of the molecular beacon in MB-LAMP

**Figure 2:**
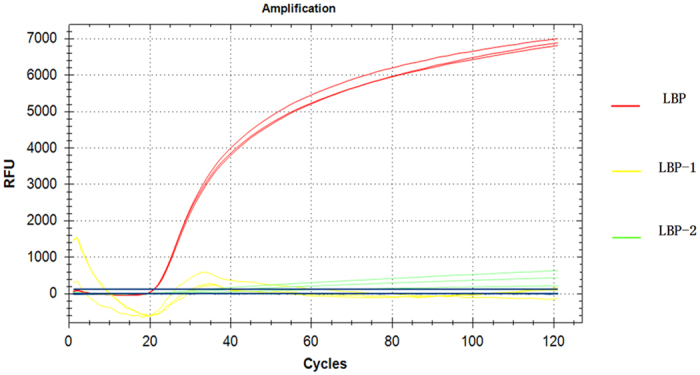
Fluorescence of different-length molecular beacons in LAMP. Fluorescence values of 3-bp and 23-bp loop neck MBs remained undetectable and around the baseline. The fluorescence of the optimal structure (13-bp loop neck and 6-bp arm), achieved a significantly detectable 7000 RFU (relative fluorescence units) in the reaction.

To monitor LAMP reactions successfully, molecular beacons must hybridize to their targets at the isothermal temperature suitable for the reaction, which can be achieved through selection of the appropriate length of the probe and arm sequences. In PCR, the length of the probe sequence is usually 15–30 nucleotides and the length of the arm sequence is 5–7 nucleotides. In our experiment, we aimed to explore the appropriate MB length under isothermic conditions. We examined lengths of 3–23 nucleotides in our tests as the head and neck sequences and found that both overly short and overly long MBs were unusable as a validation tool for LAMP. We selected the extreme 3 bp and 23 bp lengths and the common 13 bp length for head and neck sequences for evaluation; the nucleic acid sequences are listed at Table 1. Longer loop sequences have some advantages in ensuring that probe-target hybrids containing mismatches are stable, which is important for the detection of bacterial subtypes exhibiting one or two nucleotide substitutions^14^. However, the extra length reduces combination efficiency with the template and encourages the formation of secondary structures^15^. Shorter loop sequences reduce detection sensitivity, and the decreased distance between the fluorophore and the quencher increases the likelihood of unintended quenching. Ultimately, we determined that the ideal lengths of the probe and arm sequences were 25 bp and 6 bp, respectively, as these lengths were associated with high-intensity fluorescence as shown in Fig. 2. As we did not know the best concentration and temperature of the reaction at the time, the traditional concentration and temperature of 3.2 × 10^−4^ mM and 65 °C were used in the probe selection experiments.

### Optimal molecular beacon concentrations in MB-LAMP

**Figure 3:**
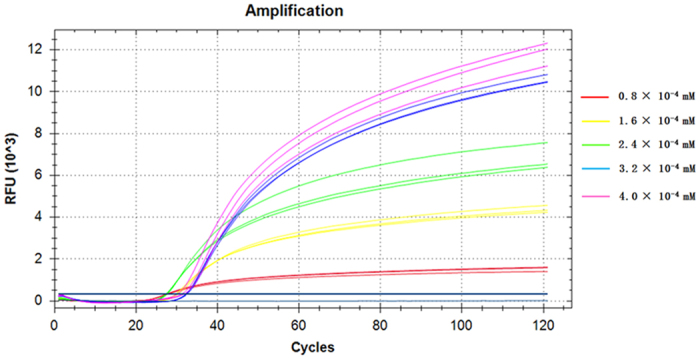
The fluorescence intensity at different concentrations of molecular beacon probes. Probe concentrations corresponding to the five cluster lines, from bottom to top, were: 0.8 × 10^−4^ mM, 1.6 × 10^−4^ mM, 2.4 × 10^−4^ mM, 3.2 × 10^−4^ mM, and 4.0 × 10^−4^ mM. The second highest concentration (3.2 × 10^−4^ mM) was selected as optimal.

The standard MB concentration in traditional quantitative PCR (qPCR) is approximately 3.2 × 10^−4^ mM. To determine optimal MB concentrations for MB-LAMP, we tested five concentrations in our assay: 0.8 × 10^−4^ mM, 1.6 × 10^−4^ mM, 2.4 × 10^−4^ mM, 3.2 × 10^−4^ mM, and 4.0 × 10^−4^ mM (corresponding to 2 pmol, 4 pmol, 6 pmol, 8 pmol, and 10 pmol). After several experiments, we found that fluorescence intensity increased significantly with increases in probe concentration until a probe concentration of 3.2 × 10^−4^ mM, and no real difference was visible between the two highest MB concentrations (Fig. 3). We thus selected 3.2 × 10^−4^ mM as the optimal MB concentration for MB-LAMP. These experiments were performed at a temperature of 65 °C.

### Reaction temperature of MB-LAMP

**Figure 4:**
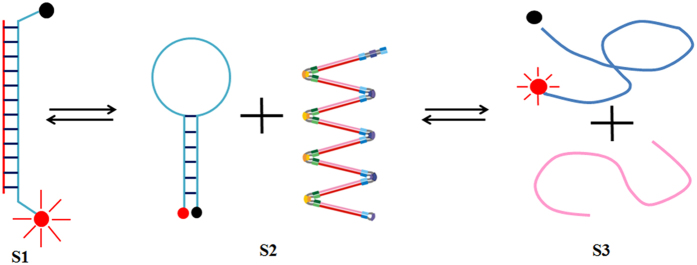
The configurations of molecular beacon (MB) under different temperature. S2 shows a free-state molecular beacon; S1 shows the hybrid state which produce the fluorescence; When the temperature is too high, the double chains will be dissociated and form the state of S3.

**Figure 5:**
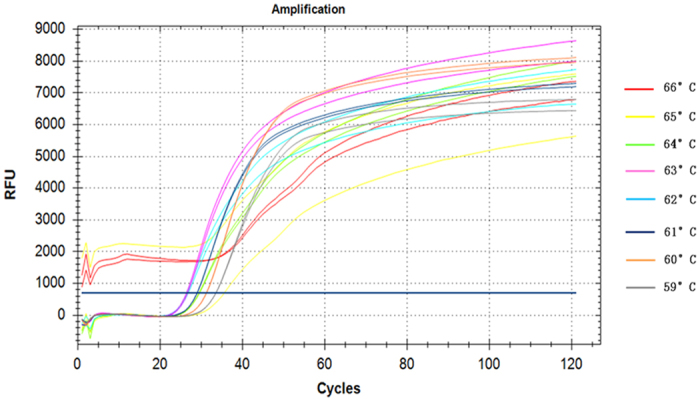
Amplification curves of MB-LAMP at different temperatures. In conjunction with Fig. 4, the results depicted demonstrate that 63 °C was the ideal temperature within the tested range of 60–66 °C.

The temperature range of LAMP is approximately 60–69 °C, with an optimum temperature of approximately 65 °C. The temperature strongly influences MB fluorescence intensity^16^ (Fig. 4). As shown in Fig. 4, S2 shows a free-state molecular beacon with a typical hairpin structure that has no fluorescence. At an appropriate reaction temperature, S2 converts dynamically to S1 and S3. Temperature optimization serves to achieve the S1 state, preventing the complete separation of the double-stranded DNA template while pushing the MB out of its hairpin conformation. Therefore, it was necessary to optimize the reaction temperature for maximum fluorescence and LAMP efficiency at the appropriate probe concentration. Our results demonstrated that 63 °C was the ideal temperature within the tested range of 60–66 °C (Fig. 5). In the 65–66 °C range, the amplification curve was unstable and the baseline remained above the threshold. Between 59 and 64 °C, the Ct (cycle threshold; x-axis) value of the amplification curves increased after an initial decrease. The temperature of 63 °C provided the lowest Ct number (26 cycles) and maximum amplification efficiency in this experiment. However, because the different combinations of MBs and primers are more easily affected by the temperature, 63 °C is not necessarily the best reaction temperature for all MB-LAMP reactions. These experiments could thus be repeated to obtain the conditions that provide the highest reaction efficiency for the detection of other pathogens.

### Specificity of MB-LAMP

**Figure 6:**
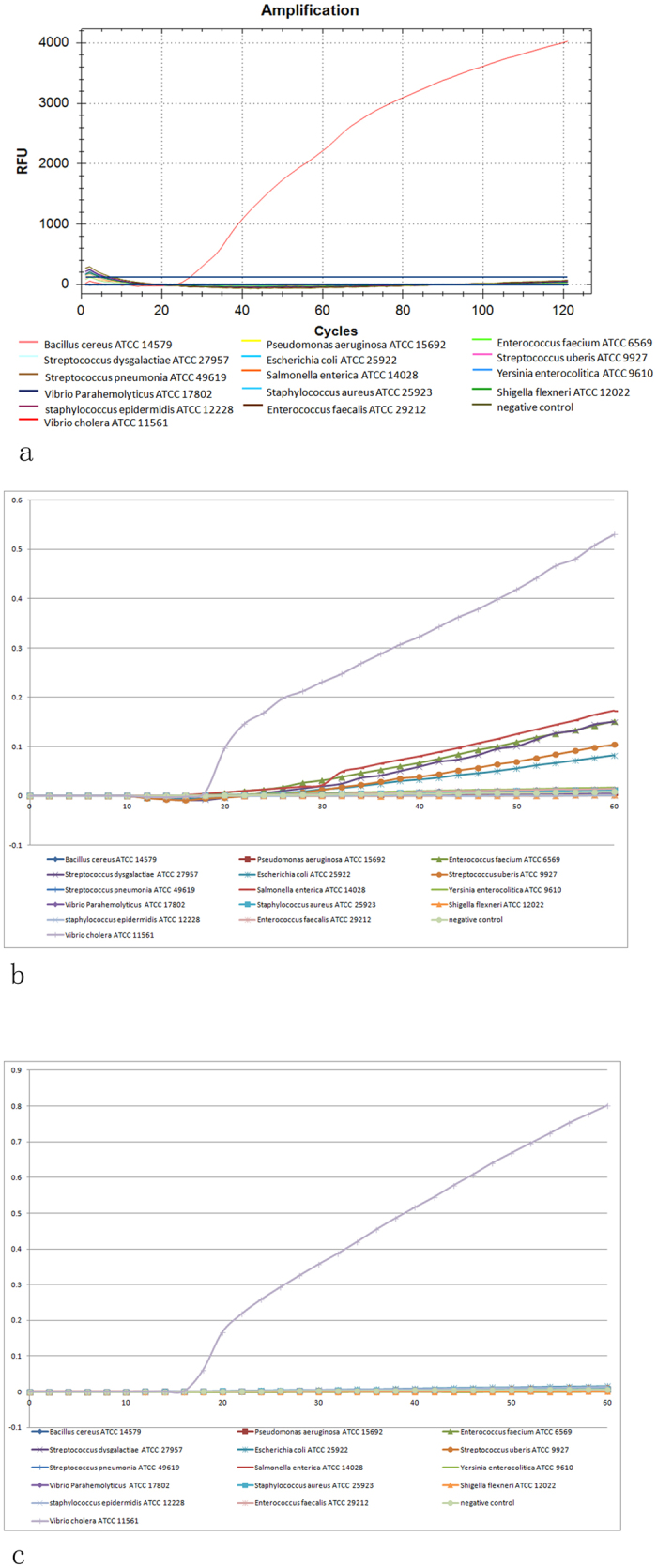
The specificity of MB-LAMP and LAMP. (**a)** The specificity of MB-LAMP. The significant increase in fluorescence was *ompW* gene and no other fluorescence were detected caused by the other strains (Table 2). It is proved this designed MB-LAMP primers were specific to the *ompW* gene. (**b**) The specificity of LAMP at the temperature of 63 °C. Although the *ompW* gene was significantly amplified, there are five atypical nonspecific reactions at 63 °C. The curves of the nonspecific amplifications rose slowly at the last stage of amplification only, which was different from the standard amplification curve. (**c)** The specificity of LAMP at the temperature of 65 °C. When the temperature reached 65 °C, only the *ompW* gene was significantly amplified. The higher temperature may affect the combination between the primers and avail the specificity of LAMP amplification.

The standard remedy for nonspecific amplification in LAMP is to verify primer specificity individually using a blank control. Because MB is highly specific, its use greatly improves the detection specificity in a LAMP reaction. Figure 6a shows that the *ompW* gene was significantly amplified at Ct = 28, or 14 min. The other standard strains (Table 2) and the blank control remained below the threshold (straight horizontal line) for the entire experiment. Figure 6b shows five atypical nonspecific reactions at the last stage of LAMP amplification at 63 °C; this problem was solved when the reaction temperature was adjusted to 65 °C (Fig. 6c). This type of curve may indicate primer dimerization rather than actual amplification of the target gene.

### Sensitivity of MB-LAMP

**Figure 7:**
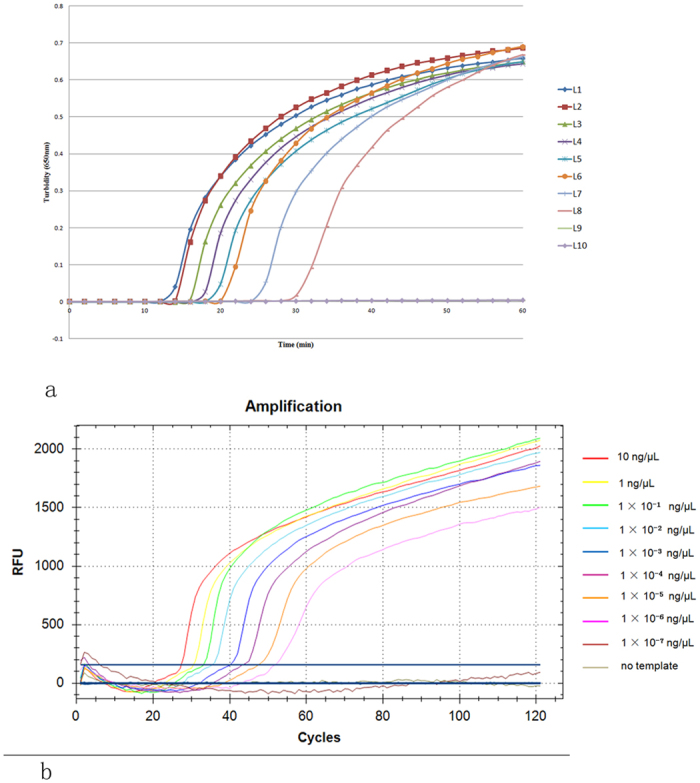
The sensitivity of LAMP and MB-LAMP to amplify DNA. (**a)** The sensitivity of LAMP Plasmid concentrations in L1 to L9 were 10 ng/μL, 1 ng/μL, 1 × 10^−1^ ng/μL, 1 × 10^−2^ ng/μL, 1 × 10^−3^ ng/μL, 1 × 10^−4^ ng/μL, 1 × 10^−5^ ng/μL, 1 × 10^−6^ ng/μL, and 1 × 10^−7^ ng/μL, whereas L10 had no template. The lower limit of detection was 1 × 10^−6^ ng/μL. (**b)** The sensitivity of MB-LAMP. The fluorescence from 8 different concentrations of DNA were detected by MB-LAMP. The DNA template concentrations were the same as those described in Fig. 7a, and the detection lower limit was also 1 × 10^−6^ ng/μL, which comes from the rightmost line.

A major advantage of LAMP is its high sensitivity (generally 10–1000 times greater than traditional PCR). To test the sensitivity of the technique, we serially diluted the plasmid from 10 ng/μL to 1 × 10^−7^ ng/μL and used distilled water as a negative control. Our results show that the reaction sensitivity of MB-LAMP was consistent with that of ordinary LAMP (Fig. 7a,b), in which the lower limit is 1 × 10^−6^ ng/μL.

## Discussion

The convenience of LAMP has increased its popularity in fields requiring rapid gene detection, but the indirect detection methods commonly employed with this technique lead to difficulty in identifying nonspecific amplification. Although all amplification methods are subject to possible contamination that can lead to false positives, nonspecific amplification is a particular problem with LAMP because of the greater sensitivity associated with an increased number of primers. To provide convincing data, satisfactory LAMP results have to be obtained by screening and designing primers or increasing the reaction temperature, as we did in these experiments. In this study, we developed and validated the use of MBs in conjunction with LAMP. Because MBs can directly detect and bind to amplification products, our detection method performs better than traditional LAMP in terms of specificity.

In addition to avoiding false positives caused by nonspecific amplification, the short reaction time and low equipment costs associated with isothermal amplification suggest that MB-LAMP may have a similar impact to the introduction of quantitative PCR in the future. Numerous inexpensive and small isothermic fluorescence detectors are widely available (e.g., gene 2, tuber scanner, min 8). Attempts to pair fluorescent probes with isothermal amplification methods have been previously successful, such as the use of fluorescence energy transfer probes with strand displacement amplification (SDA), molecular beacons with nucleic acid sequence-based amplification (NASBA), and padlock probes with rolling circle amplification (RCA)^17^,^18^,^19^. Based on these techniques and the outcome of the current experiments, MB-LAMP could also be extended to most isothermal amplification methods, such as polymerase spiral reaction (PSR)^20^, cross-priming amplification(CPA)^21^, transcript-based amplification system (TAS)^22^, and single primer isothermal amplification (SPIA)^23^.

The promise of MB-LAMP as a widespread diagnostic tool was recently demonstrated by its successful use to test for the Zika virus in our laboratory (Supplementary Information). The MB-LAMP technology still requires evaluation to understand mechanistic aspects, e.g., why the curve of LBP-1 slightly increases before the overall fluorescence intensity becomes flat as the number of cycles increase, as shown in Fig. 2. Additionally, there is still some room for improvement in MB-LAMP, such as in the design and selection of probe. In the present study, we designed the probe artificially, as there is no specific software to design the probe sequence to match the loop primers. However, there have been many studies on MB structural dynamics and composition that varied the loop length and stem length^15^,^16^. We believe that with further development of such studies, the design process for MBs will become more simple and uniform. With the expected increase in the utilization of MB-LAMP, the coordinated development of MB and MB-LAMP will enable an optimal combination of the MB design and MB-LAMP characteristics. In addition, we believe that MB-LAMP could eventually be a quantitative analysis method, similar to real-time PCR. A standard curve could be obtained according to the Ct values of different concentrations of a standard template, thus enabling calculation of the concentration in the sample. The validity of such methods is dependent on amplification cycle number and the accuracy of fluorescent detection; we therefore propose that future work focus on the development of quantitative LAMP procedures. Although the establishment of quantitative MB-LAMP still requires considerable research, we are confident that continual work will further stabilize and improve this method. The development of MB-LAMP should contribute greatly to DNA detection and quantification.

## Methods

### DNA preparation

Genomic DNA of several bacterial strains (Table 2) was extracted with the TIANamp Bacteria DNA Kit (Tiangen Biotech Co., Ltd., Beijing, China) following the manufacturer’s protocol, and samples were stored at −20 °C until use as templates in the LAMP and MB-LAMP assays.

### Samples

We used MB-LAMP to detect a part of the 1031-bp *ompW* gene from *Vibrio cholerae* (GenBank: X51948.1). A 222-bp synthesized target plasmid of the *ompW* gene (Supplementary Information) was quantified using Qubit 3.0 (Life Technologies, Carlsbad, CA, USA). We serially diluted the plasmid to 1 × 10^−8^ ng/μL in ddH_2_O for use as templates.

### Primers and molecular beacon probes

**Figure 8:**
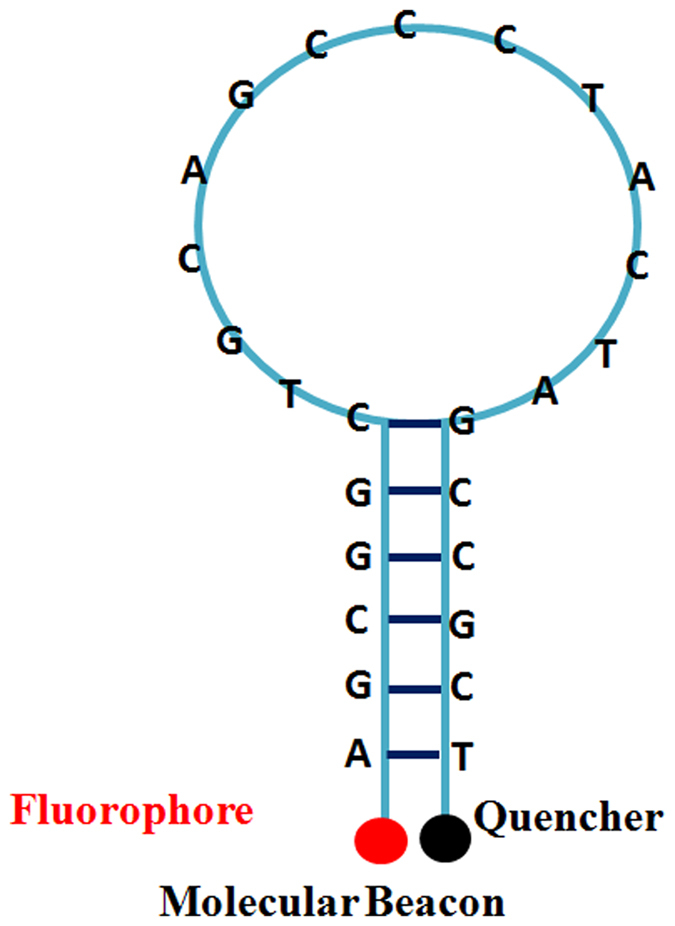
The transformation of LB loop primers to the molecular beacon (MB). The fluorescent nucleic-acid probe LFP LBP were modified from the LF LB as described in text, respectively.

The LAMP primers (F3: outer forward primer; B3: outer backward primer; FIP: forward inner primer; BIP: backward inner primer) were designed in Primer Explorer V4 (http:/primerexplorer.jp/lamp). Two additional loop primers (LF: loop F, LB: loop B) were designed to accelerate amplification. The fluorescent nucleic-acid probes in MB-LAMP (LBP and LFP) were directly modified from LB and LF (Fig. 8). As shown in Fig. 8, the fluorescent nucleic-acid probe LBP was modified from the LB; the last 19 nucleotides in the LBP sequence are identical to the LB and form the heads as well as one arm of the molecular beacon. The first six sequences of the LBP are the reverse complement of the last six sequences and form the other MB arm. The fluorophore and quencher are linked to each MB arm, respectively. The LFP was transformed from the LF in the same way. We chose the pair of LBP and LF in our MB-LAMP experiment randomly. Table 3 shows the LAMP primers and molecular beacon probes used in this manuscript. Table 1 shows the long and short molecular beacon probe sequences used in the experiment for MB length optimization (Fig. 2). One of the arms and the head of LBP-1 were the extended sequences of LB, which was part of the *ompW* gene. We only needed to use the 29-base sequence from the LB and to mark the last six bases and their reverse complementary sequences as arms. Finally, we connected the fluorescence and quenching group. Similarity, one of the arms and the head of LBP-2 is a part of LB. We used the first 9-base sequence from the LB and marked the last six bases and their reverse complementary sequences as arms. After connection of the fluorescence and quenching groups, LBP-2 was designed well.

### Reaction mixture and conditions

The reaction mixture (DNA Amplification Kit; Eiken Chemical Co., Ltd., Tochigi, Japan) for LAMP and MB-LAMP were 25 μL per reaction tube (Eiken Chemical Co., Ltd., Tochigi, Japan), containing the following reagents (final concentration): 20 mM Tris-HCl (pH = 8.8), 10 mM KCl, 10 mM (NH4)_2_SO_4_, 0.1% Tween 20, 0.8 M betaine, 8 mM MgSO_4_, 1.4 mM each dNTP, and 8 U Bst DNA polymerase. Primer concentrations for LAMP were 40 pmol of FIP and BIP, 20 pmol of LF, and 5 pmol of F3 and B3; concentrations for MB-LAMP were 40 pmol of FIP and BIP, 20 pmol of LF, 2–10 pmol of LBP, and 5 pmol of F3 and B3. In addition, template genomic DNA (2 μL) was added. The LAMP was performed at the traditional temperature of 65 °C, and the optimum conditions for MB-LAMP were determined in the experiments described in this manuscript. We used the concentration of 3.2 × 10^−4^ mM and temperature of 63 °C once they were established as the optimal conditions.

### Detection for LAMP and MB-LAMP

Turbidity in the LAMP assay was monitored using a Loop Amp real-time turbidimeter (LA-320c; Eiken Chemical Co., Ltd.), and the OD was recorded at 650 nm in 6-s intervals. Fluorescence of MB-LAMP was monitored with a CFX Connect Real-Time PCR Detection System (Bio-Rad, Hercules, CA, USA) and amplifications were performed under 120 cycles of constant temperature for 30 s. The fluorescence value was read in 30-s (one cycle) intervals.

## Additional Information

**How to cite this article**: Liu, W. *et al*. Establishment of an accurate and fast detection method using molecular beacons in loop-mediated isothermal amplification assay. *Sci. Rep.* **7**, 40125; doi: 10.1038/srep40125 (2017).

**Publisher's note:** Springer Nature remains neutral with regard to jurisdictional claims in published maps and institutional affiliations.

## Supplementary Material

Supplementary Information

## Figures and Tables

**Figure 1:**
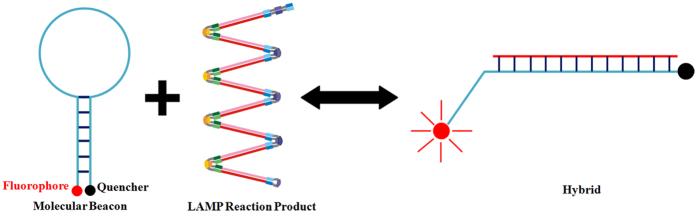
Schematic of molecular-beacon action. Molecular beacons are single-chain oligo nucleotides with a hairpin structure, which keeps the fluorophore in close proximity to the quencher, preventing fluorescence. When the molecular-beacon probe hybridizes to its target and forms a double helix, the change in conformation separates the quencher from the fluorophore, thereby restoring fluorescence.

**Table 1:** The sequences of MBs of different length.

Primer	instruction	Sequence (5′–3′)
LBP-1	23-bp loop neck MB	FAM-CAAATATGCAGCCCTACTAGCCGCTCCTGTATTTG-Dabcyl
LBP-2	3-bp loop neck MB	FAM-AGGGCTTGCAGCCCT-Dabcyl
LBP	13-bp loop neck MB	FAM-AGCGGCTGCAGCCCTACTAGCCGCT-Dabcyl

**Table 2:** Bacteria used in the experiment.

Species	source
*Bacillus cereus* ATCC 14579	Our microorganism center
*Pseudomonas aeruginosa* ATCC 15692	Our microorganism center
*Enterococcus faecium* ATCC 6569	Our microorganism center
*Streptococcus dysgalactiae* ATCC 27957	Our microorganism center
*Escherichia coli* ATCC 25922	Our microorganism center
*Streptococcus uberis* ATCC 9927	Our microorganism center
*Streptococcus pneumonia* ATCC 49619	Our microorganism center
*Salmonella enterica* ATCC 14028	Our microorganism center
*Yersinia enterocolitica* ATCC 9610	Our microorganism center
*Vibrio Parahemolyticus* ATCC 17802	Our microorganism center
*Staphylococcus aureus* ATCC 25923	Our microorganism center
*Shigella flexneri* ATCC 12022	Our microorganism center
*staphylococcus epidermidis* ATCC 12228	Our microorganism center
*Enterococcus faecalis* ATCC 29212	Our microorganism center
*Vibrio cholera* ATCC 11561	Our microorganism center

**Table 3:** Primers used for LAMP and MB-LAMP.

Primer	Type	Sequence (5′–3′)
HL-F3	forward outer	CCTAAATGTAGCAAATTGATTTCCT
HL-B3	backward outer	GTCATTAGGTACTACCGAGG
HL-FIP	forward inner	TCCTTTTTTGTAGGGCTATGTTGTTGTTTGTGTGATTTTTGTGTGC
HL-BIP	backward inner	ACCATTTGCCTAGCCGTACTACAATAAAGTCACCTTCTTGG
HL-LF	loop forward	TGTGTTGCGCGCACAGTA
HL-LB	Loop backward	TGCAGCCCTACTAGCCGCT
LBP	Loop backward primer	FAM-AGCGGCTGCAGCCCTACTAGCCGCT-Dabcyl
