# Supplementary material for: Establishment of an accurate and fast detection method using molecular beacons in loop-mediated isothermal amplification assay
Source: Sci Rep. 2017 Jan 6;7:40125. doi: 10.1038/srep40125 (PMC5216335; doi:10.1038/srep40125)
Supplement: Supplementary Information [file srep40125-s1.pdf]

Establishment of an accurate and fast detection method using molecular beacons in loop-mediated isothermal amplification assay

Wei Liu [#], Simo Huang [#], Ningwei Liu, Derong Dong, Zhan Yang, Yue Tang, Wen Ma, Xiaoming He, Da Ao, Yaqing Xu, Dayang Zou *, Liuyu Huang *

The 222-bp synthesized target plasmid of the ompW gene:

CCTAAATGTAGCAAATTGATTTCCTACAAGTTTGTGTGATTTTTGTGTGCTACTGT
GCGCGCAACACAAAGATAACAACATAGCCCTACAAAAAAGGAAAACGTCATGAA
ACAAACCATTTGCCTAGCCGTACTTGCAGCCCTACTAGCCGCTCCTGTATTTGC
TCACCAAGAAGGTGACTTTATTGTGCGCGCGGGTATTGCCTCGGTAGTACCTAA
TGAC

Target gene of Zika detection using MB-LAMP:

Zika virus strain ArD158095 polyprotein gene (ID: KF383121.1)

Supplementary Table S1. Primers used for LAMP and MB-LAMP in Zika.

Primer	Type	Sequence (5'–3')
ZK-F3	forward outer	GAAGGAGCCGTTCACACG
ZK-B3	backward outer	CCTGCATACTGCACCTCC
ZK-FIP	forward inner	GGCGGCATTTCAAATGGCCAGCTCGCTG GAGCTAGAGG

ZK-BIP	backward inner	TATTCCTTGTGCACTGCGGCATGACTGTT CCATGCAGTGTT
ZK-LF	loop forward	CCTTTGCACCATCCATCTCAG
ZK-LB	Loop backward	TTCACATTCACCAAGGTCCCA
LFP	loop forward primer	FAM- CTGAGATCCCTTTGCACCATCCATCTCAG -Dabcyl
LBP	Loop backward primer	FAM- CACCAAGGTCCCAGCTGATTGGTG -Dabcyl

Supplementary Figure S1. feasibility of MB in Zika detection

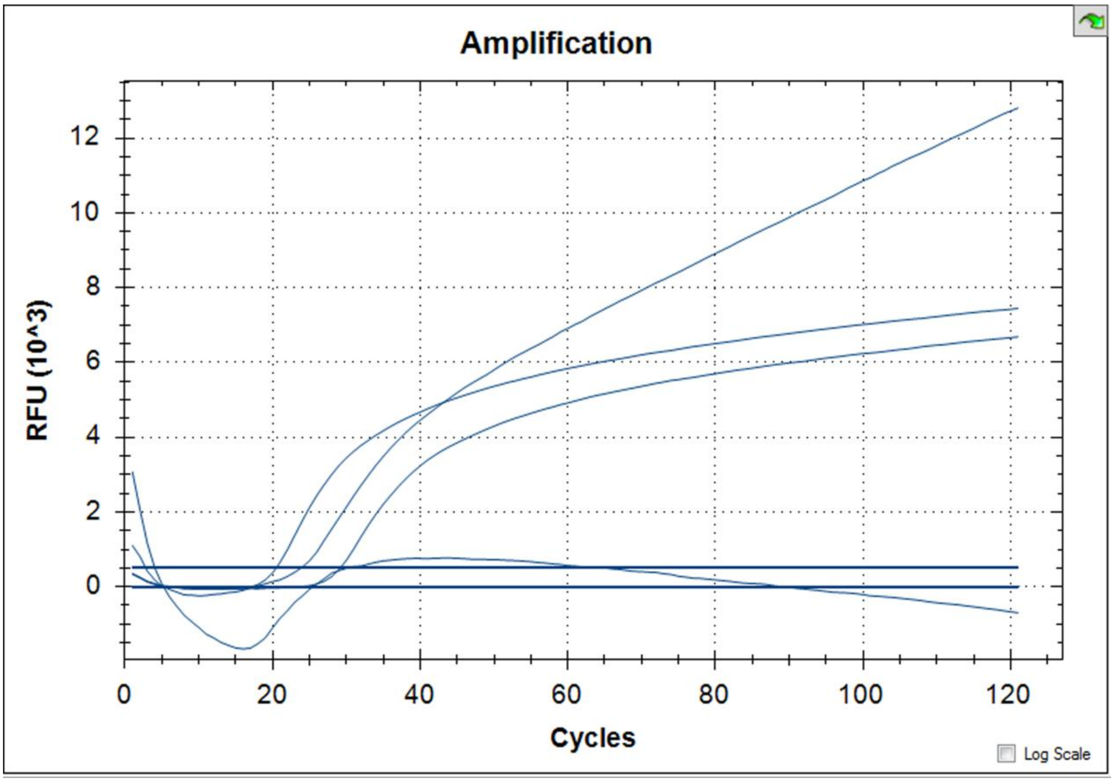

Although the data of Zika has not been published, the result is feasible. We used the same length, concentration, and temperature as described in the manuscript and obtained favourable results. The figure shows two sets of probes of Zika, LF & LBP and LB & LFP, each of them can be amplified and produce fluorescence. However, the condition has not be optimized yet, there is one curve does not completely amplified. Nevertheless the MB probe is proved to be viable in Zika too.
